# Novel convolutional neural network for bacterial identification of confocal microscopic datasets

**DOI:** 10.1038/s41598-026-38861-5

**Published:** 2026-02-10

**Authors:** Ahmed Al-Jumaili, Saif Al-Jumaili, Salam Alyassri, Adil Deniz Duru, Osman Nuri Uçan, Mohan V. Jacob, Frederico Branco, Paulo Jorge Coelho, Ivan Miguel Pires

**Affiliations:** 1 Electronics Materials Lab, College of Science and Engineering, James Cook University, Townsville, QLD 4811 Australia; 2 Department of Medical Physics, College of Applied Science, University of Fallujah, Al-Anbar, 31002 Iraq; 3 Institute of Biophysics and Biomedical Engineering (IBEB), Faculty of Sciences, University of Lisbon, Lisbon, Portugal; 4 AquaValor-Centro de Valorização e Transferência de Tecnologia da Água, 5400-342 Chaves, Portugal; 5 Department of Electrical and Computer Engineering, Altinbas University, Istanbul, Turkey; 6 Faculty of Sport Sciences, Sports Coaching Education, Marmara University, Istanbul, Turkey; 7 Institute for Systems and Computer Engineering, Technology and Science (INESC TEC), 4200-465 Porto, Portugal; 8 School of Science and Technology, Universidade de Trás-Os-Montes e Alto Douro, 5000-801 Vila Real, Portugal; 9 Institute for Systems Engineering and Computers at Coimbra (INESC Coimbra), 3030-290 Coimbra, Portugal; 10 School of Technology and Management, Polytechnic of Leiria, 2411-901 Leiria, Portugal; 11 Instituto de Telecomunicações, Escola Sperior de Tecnologia e Gestão de Águeda, Universidade de Aveiro, Águeda, Portugal

**Keywords:** Artificial intelligence, CNNs, Bacterial classification, Confocal microscopy, Computational biology and bioinformatics, Mathematics and computing

## Abstract

Artificial intelligence (AI), complex mathematical algorithms, is currently employed across various fields to perform tasks quickly and effectively. In this study, a novel deep-learning algorithm named (CM-Net) was developed to classify biological data obtained as images from Confocal Microscopy. The images were collected for two types of bacterial species: (*Escherichia coli* and *Staphylococcus aureus*), where the number of images was 300 for each class. To enhance the dataset, we divided each image (using the augmentation method) into a small number of images with 224 × 224 dimensions, resulting in a total of 7066 images for both classes. These augmented images were fed to CM-Net to ensure accurate results and avoid bias in the developed algorithms. The algorithm was trained and tested 30 times with a 5-K cross-validation for each time. The algorithm’s performance was evaluated using seven metrics (accuracy, sensitivity, specificity, precision, NVA, F1-score, and MCC), where the respective results were 96.08%, 95.98%, 96.19%, 96.78%, 95.26%, 96.38%, and 92.11%, indicating the model’s high accuracy and reliability. CM-Net drastically reduces bacterial identification time by automating large-scale data analysis, processing results in 8.9 min. The automation provided by CM-Net simplifies workflows, enabling non-expert workers to perform microbial identification without extensive training. The significant outcomes of applying CM-Net for bacterial identification revolve around its transformative impact on data analysis’s speed, efficiency, and accuracy, making advanced analysis accessible to non-experts while minimizing human error.

## Introduction

Artificial intelligence (AI), specifically deep learning techniques, is a branch of computer science where complex mathematical algorithms are employed to create innovative tools to perform tasks quickly and efficiently^1^. Convolutional Neural Networks (CNNs) are a specialized type of deep learning designed for handing out structured grid-like 2-dimensional data types^2^. CNNs’ algorithms attempt to mimic how the human brain processes information by analyzing specific patterns in large datasets^3^. CNNs involve several layers, including the Input Layer, Convolutional Layer^4^, Pooling Layer^5^, Activation Function^6^, and SoftMax Layer^7^, where each layer has a specific role in the feature extraction and learning model. The current trend of deep technique learning empowers machines to learn and make decisions without explicit programming. For instance, the well-designed model distinguishes between dissimilar items by examining features like edges, colors, and textures in image classification.

Artificial intelligence has rapidly become an important part of current healthcare systems and professional humans’ ongoing research arena. In microbiology, identifying bacterial species is crucial for medical diagnostics^8^, treatment procedures^9^, preventing pathogenic infections^10^, cell counting^11^, and ensuring food security^12^. Here, CNN is preferably suited for training algorithms to differentiate between various microorganisms, considering many factors such as morphological features^13^, color, and growth patterns^14^. Using those trained algorithms, identifying different types of bacteria can be faster and more accurate. The process is achieved by simply applying microscopic images to pre-trained CNN algorithms.

Reports have shown that researchers applied CNN models on various imaging data obtained from currently available microscopes, such as optical microscopy^15^, scanning electronic microscopy^16^, fluorescence microscopy^17^, etc. Mitsuko Hayashi et al. performed a morphological analysis of *E*. *coli*. microscope images using transmission electron microscopy (TEM), identifying the strains based on the classification outcomes^18,19^. Yanan Zhu et al. applied convolutional neural network approaches to single-particle cryo-electron microscopy (cryo-EM) images of biological molecules, enabling computerized selection and extraction of particles from raw cryo-EM micrographs^20^. The segmentation of SEM images has been reported using deep convolutional neural networks to separate bacterial cells from their cluster, estimating the number of individual cells, length, and width of their distributions^21^. Further researchers applied lens-free microscopy imaging to train neural networks that can detect and classify pathogenic species based on the time-lapse growth of bacteria on thin-layer agar plates^22^. Kang et al. applied convolutional neural networks to hyperspectral microscope images to develop an optical diagnostic tool for detecting and classifying various foodborne bacteria at the cellular level^23^. Likewise, Chenglong et al. built an AI-assisted system for bacteria genus identification by combining hyperspectral microscopic technology and deep-learning-based algorithms, which take 30 s to classify a pathogen^24^.

Among available explorative microscopies, a confocal laser scanning microscope (CLSM) is an advanced optical microscope that focuses a laser beam on a sample. The laser hits fluorescing molecules, often a staining fluorescent dye, where these molecules emit a spectrum that can be used to determine different chemical components and imaging of a given sample. CLSM allows focusing on a small focal plane within a sample, where Z-control-apparatus governs precise depth movements (∼ 10 nm) in the axial plane of the sample. Images are obtained point-by-point as a 2D micrograph, then reconstructed as high-resolution 3D with depth selectivity images. One valuable application of CSLM in bacteriology is to visualize microbes and distinguish between live and dead bacterial cells in the biofilm matrix. Dead cells appear red (indicating compromised membranes), while living cells appear green, revealing intact membranes. The interrupting CLSM images require time, resources, and experts to adjust background noise, tune photobleaching, and use functions to threshold the signal-to-noise ratio. In addition, untreated cells or non-fluorescent dye should be separated/ignored to validate staining specificity. Here, deep learning algorithms can be adapted to aid researchers in effectively processing acquired CSLM micro visualization. Algorithms can handle large, complex datasets, such as identifying the bacterial type and recognizing dead/live cells.

This article reports a novel convolutional neural structure for recognizing dead/live bacteria and classifying microbial types for CLSM images in a one-step method. The developed algorithms involve a convolution layer, a batch normalization layer, a clipped ReLU (Rectified Linear Unit) layer, and a max-pooling layer. We obtained original CLSM images for two types of classical bacteria, namely *E.coli* and *S. aureus*, in the PC2-microbiological lab. Further, the data augmentation technique is used to artificially expand the size of the dataset by generating new data points from the original data, where large data are vital for training robust algorithms. Data augmentation is principally applied to enhance the accuracy of the developed software by presenting variability, minimizing overfitting, and simulating dissimilar scenarios. To the best of our knowledge, no one-model deep convolutional neural network was developed to automatically distinguish dead and live bacterium and identify microbial cell nature for CLSM data sets.

## Dataset

### Bacterial growth and confocal microscopy imaging

**Fig. 1:**
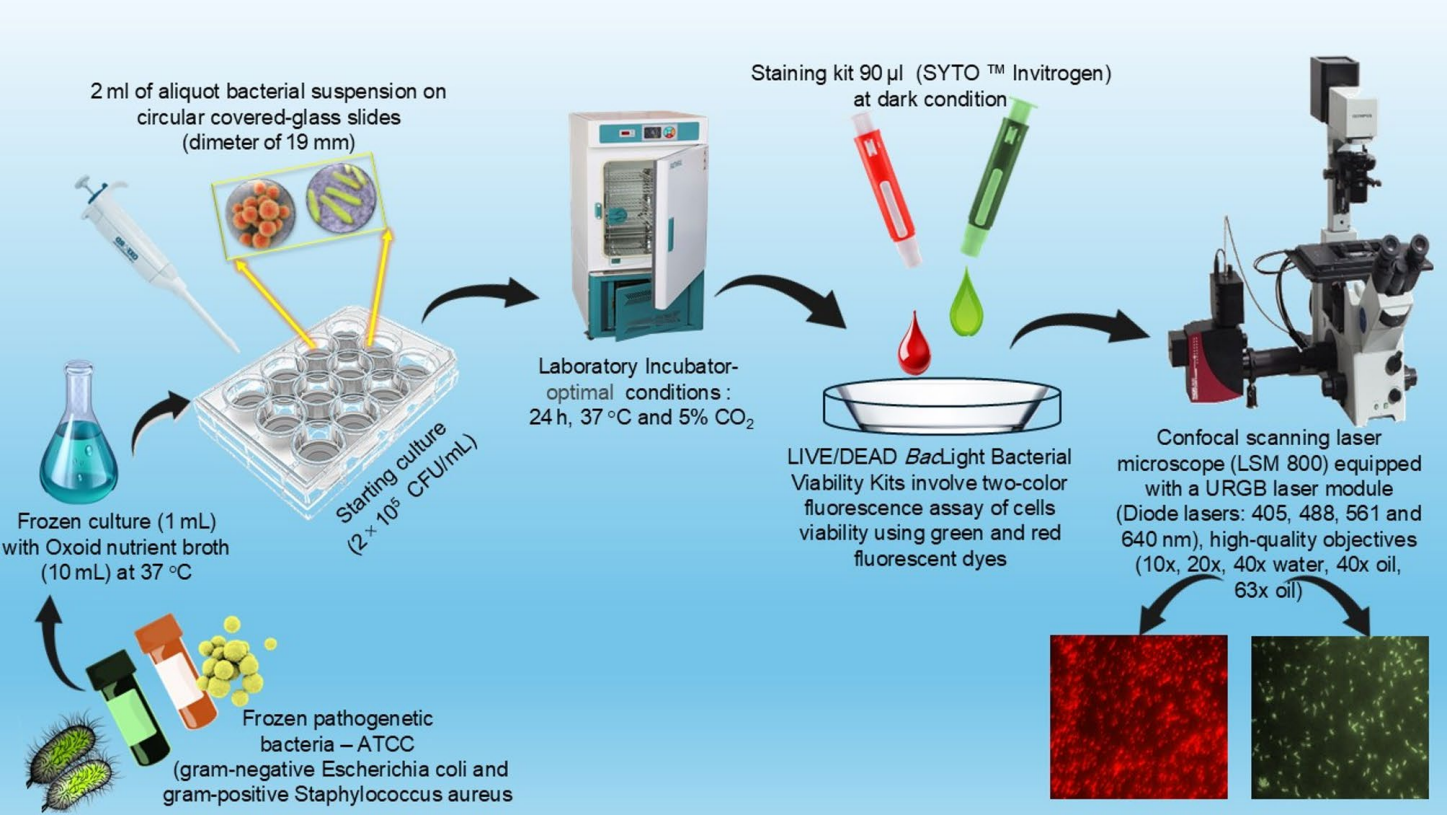
A representation shows the procedure of bacterial growth and confocal microscopy imaging.

The bacterial studies were achieved using standard pathogens (gram-negative *Escherichia coli* and gram-positive *Staphylococcus aureus*) obtained from the American Type Culture Collection (ATCC). These microorganisms are well-known to cause infections in healthcare facilities and implantable devices^25^. To experiment In-vitro, a bacterial suspension was made by mixing the frozen standard culture (1 mL) with typical Oxoid nutrient broth (10 mL) at 37 °C, then thoroughly shaken at 120 rpm. A spectrometer device (The SPECTROstar Nano, BMG lab tech, Germany) was used to precisely evaluate several bacterial cells in the suspension before being placed on the surface of a sterilized glass slide. The microbial cell density was used to be (OD600 = 0.1) to achieve a consistent starting culture (2 × 10^5^ CFU/mL). Circular covered-glass slides (diameter of 19 mm) were sited into typical 12-well dishes, then an aliquot bacterial suspension (2 ml) at a concentration of 2 × 10^5^ CFU/mL was carefully poured onto the slide surface. At that point, all slides were incubated at standard conditions (24 h, 37 °C, and 5% CO_2_) to get proper growth of microbial cells. All experimentation was run in triplicate to ensure accuracy. Figure 1 presents the representation of the procedure for of bacterial growth and confocal microscopy imaging.

After incubation, a staining kit (*SYTO™* Invitrogen, Thermo Fisher, USA) was employed to evaluate live and dead bacterial cells. The dyes were applied following the protocol from a supplied company. Microbial suspensions were gently removed from the slides using pipettes to prepare samples for imaging. Then, 90 µl of the staining kit was poured on each slide and stored in a lab case box to keep the sample for 20 min in complete darkness. Then, slides were carefully splashed with purified water to remove unattached bacterial cells. The slides were then quickly introduced to a confocal scanning laser microscope (LSM 800, ZEISS, Germany) for imaging purposes. LIVE/DEAD Bacterial Viability Kit, for microscopy and quantitative assessment, are fluorescent staining components typically used for evaluating the viability of microbial cells. It allows a two-color fluorescence test of microbial viability by displaying green and red luminous dyes for microscopy imaging. The kit enables microbiologists to distinguish between live and dead bacterial cells within a short time (a few minutes) through fluorescent imaging. In the fluorescence technique, the live bacterial cells appear green and dead cells appear red, where cells are separately viewed.

**Fig. 2:**
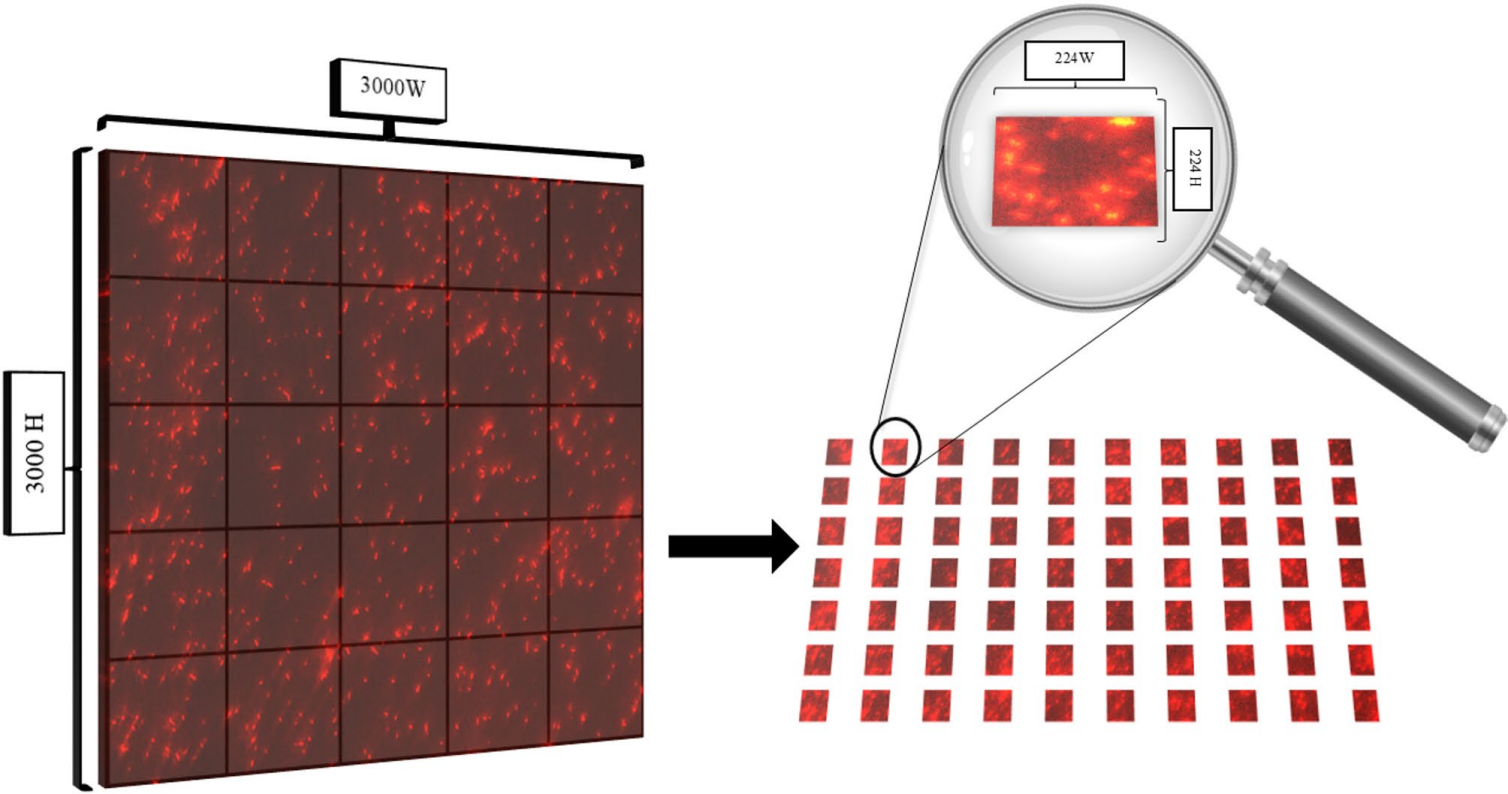
An illustration of the augmentation method used to divide images into parts to obtain many sub-images (patches).

Taking images with a laser microscope is one of the most challenging things because many procedures must be taken to obtain enough images to develop a model to determine the type of bacteria. Therefore, the number of images was more than 300 for each class. The dimensions were large, measuring 3000 × 3000 pixels, as using these dimensions in training consumes a significant amount of time and the amount of RAM required for training and testing. Thus, we divided the images into dimensional blocks to ensure a robust dataset and obtain sufficient images for training and testing. The dimensions in the block for each image were 224 × 224, as these images will increase the number of images to more than 3000 images for each class and. thus this increase will positively affect the training of the model, as it will provide a more comprehensive and diverse set of samples for bacteria, which works to increase the model’s ability to classify more accurately. Figure 2 illustrates the division method used in this article.

##  Methods

### CNN-design

Deep learning, a subfield of artificial intelligence, has entirely transformed the examination of image data by greatly accelerating its processing compared to conventional approaches. Its quick analytical capabilities are particularly advantageous in microscopic domains, where deep learning expressively improves microscope performance by facilitating automated recognition, classification, and detection of bacterial species. Implementing deep learning techniques within such domains provides a significant benefit, enabling greater efficiency and precise bacterial analysis.

Although pre-trained techniques can be beneficial for categorizing microorganisms, such models are taught/trained by image datasets with little specificity toward microbiological images. Hence, their achievement in classifying bacteria might turn out less than ideal. To tackle this difficulty, we created an innovative advanced deep learning algorithm from the ground, specially intended to classify microorganisms. The model’s structure is carefully designed, including five convolutional blocks, every specifically trained to extract substantial properties via the input images that greatly enhance the end-product classification result.

**Fig. 3:**
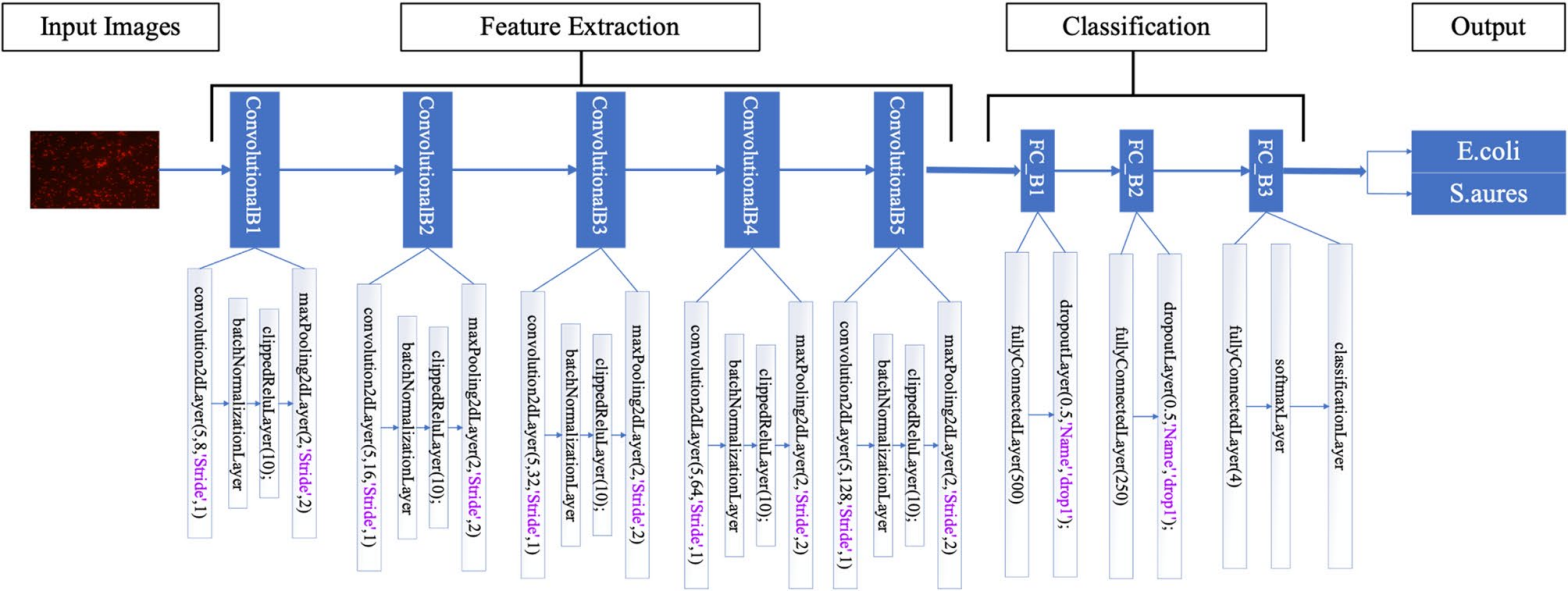
Proposed CNN, architectures, and the number of layers used inside each block.

Within the designed model, all blocks consist of a convolution layer, a batch normalization layer, a clipped ReLU (Rectified Linear Unit) layer, and a max-pooling layer. The convolution layers are specifically designed to filter the images to extract crucial patterns, whereas the batch normalization layers stabilize the learning process. Including clipped ReLU layers in the model introduces non-linearity, thus facilitating the learning of intricate characteristics. Additionally, the max-pooling layers serve to decrease the spatial dimensions, selectively emphasizing the most relevant features. After the convolutional blocks, our model contains two fully connected (FC) blocks, consisting of convolutional and dropout tiers. These layers were provided to improve the retrieved features and implement regularization to avoid overfitting. The last block is specifically allocated for the classification process, during which the features are later detected along with classified data. This block comprises fully linked layers, a softmax layer handling probability distribution, and a classification layer responsible for assigning class labels. Implementing several layers and filters in the model significantly improves its capacity to extract the most required characteristics throughout the convolutional operation. The thorough extraction of features is essential for attaining a high level of accuracy in the classification of microorganisms. As shown in Fig. 3, the architecture underlying our model showcases its efficacy in training and classifying microbiological species, providing an accurate approach for automated microscopic analysis.

Algorithm 1 presents a comprehensive pseudo-code for the convolutional neural network (CNN) model specifically developed for classifying microscopic bacterium datasets. The CNN model employs a systematic methodology for evaluating incoming data and identifying pertinent traits that may accurately differentiate among distinct bacterium kinds. The architecture comprises multiple convolutional layers containing suitable filters, succeeded by activation functions, pooling layers, and fully connected layers to enhance the acquired representations systematically. The model structure was developed to identify variations and attributes for obtaining excellent classification precision. This CNN model effectively analyzes and classifies microscopic bacterium images through an accurate and effective learning process.

The major contribution:

- A streamlined, efficient backbone utilizing ClippedReLU to prevent high activation values, hence minimizing reliance on complex structures (residuals, inception modules, depthwise separable convolutions). The simple design further decreases bias, resulting in enhanced interpretability and flexibility.
- Batch Normalization, applied following each convolution, standardizes activations, reduces inner covariate shift, and enhances convergence, resulting in increased training reliability despite the additional structural complexity.
- A conventional convolution pooling pipeline provides an effective balance between accuracy and computational efficiency, while the application of dropout following each fully connected layer provides stochastic regularization, which mitigates overfitting and enhances computational efficiency.

**Algorithm 1:**
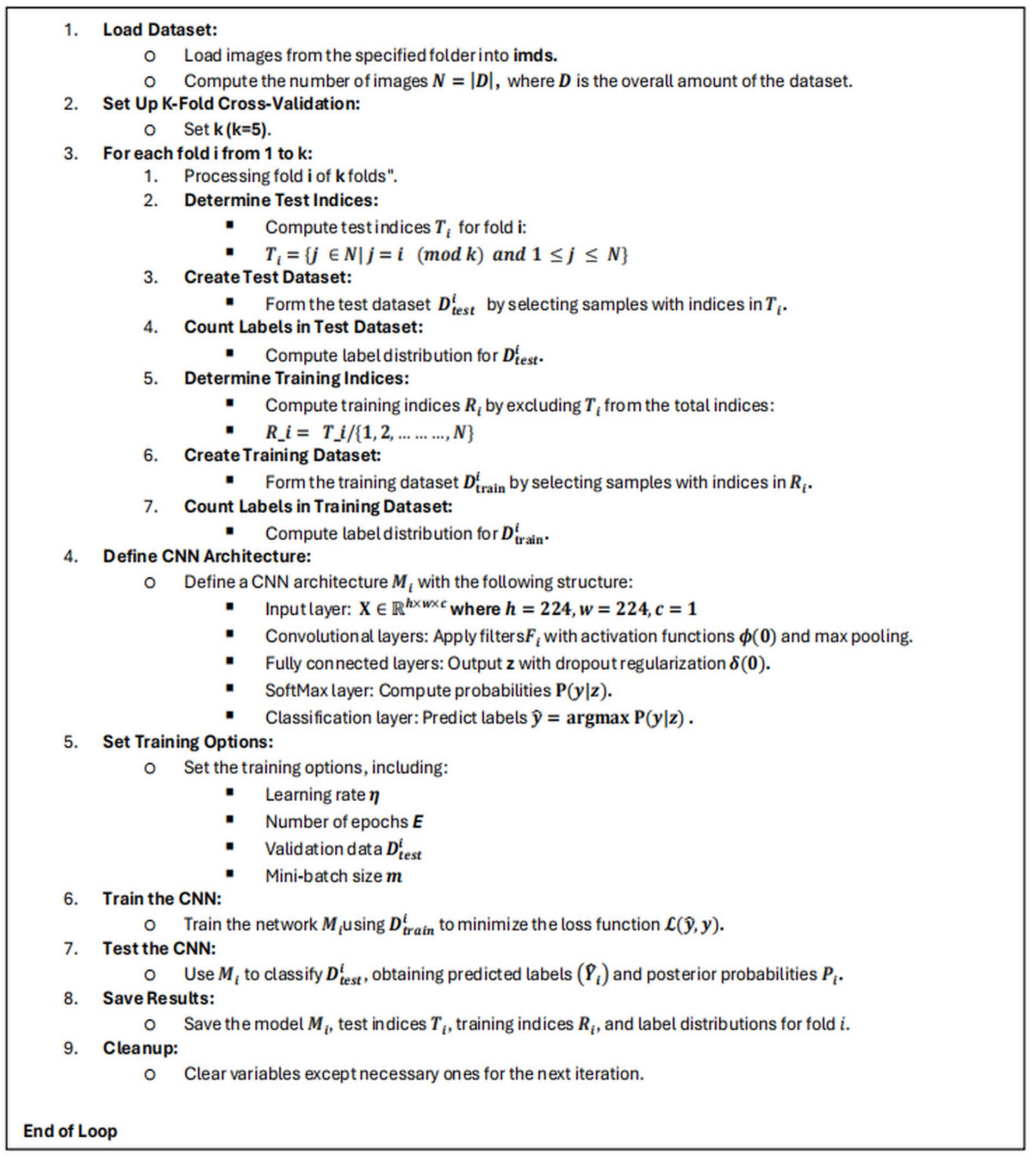
Pseudo-code for the convolutional neural network.

### CM-Net layer configuration

The architecture effectively incorporates a local-to-global inductive bias that is adept at displaying complex structural information: interspersing same-padded 3 × 3 filters with pooling enlarges the efficient receptive area to approximately 190 × 190 pixels by the final stage, allowing every activation to encapsulate almost the entire field of view while still being anchored in local texture. Implementing ClippedReLU for capping activations stabilizes optimization in small-batch contexts and produces quantitatively more stable gradients, which we determined to be beneficial for generating unambiguous Grad-CAM and occlusion-sensitivity maps focused on microbe areas. To provide a fair comparison with transfer-learning baselines, preprocessing, augmentation, optimizer/schedule, batch size, and total epochs were maintained constant; just the backbone varied. Table 1 Fully disclose layer configurations and training hyperparameters that used in CM-Net.

**Table 1:** Architectural layer configurations and training hyperparameters of the proposed convolutional neural network (CM-Net).

No.	Layer Name	Kernel Size	Stride	Channels	Activation	Pooling	Dropout	Input Size	Output Size	Parameters
1	Input	-	-	3	-	-	-	224 × 224 × 3	224 × 224 × 3	0
2	Conv_1	3 × 3	1	16	ClippedReLU(10)	MaxPool 2 × 2	-	224 × 224 × 3	112 × 112 × 16	448
3	Conv_2	3 × 3	1	16	ClippedReLU(10)	MaxPool 2 × 2	-	112 × 112 × 16	56 × 56 × 16	2,320
4	Conv_3	3 × 3	1	32	ClippedReLU(10)	MaxPool 2 × 2	-	56 × 56 × 16	28 × 28 × 32	4,640
5	Conv_4	3 × 3	1	64	ClippedReLU(10)	MaxPool 2 × 2	-	28 × 28 × 32	14 × 14 × 64	18,496
6	Conv_5	3 × 3	1	128	ClippedReLU(10)	MaxPool 2 × 2	-	14 × 14 × 64	7 × 7 × 128	73,856
7	Conv_6	3 × 3	1	256	ClippedReLU(10)	MaxPool 2 × 2	-	7 × 7 × 128	3 × 3 × 256	295,168
8	FC_1	-	-	500	-	-	0.5	3 × 3 × 256	1 × 1 × 500	1,152,500
9	FC_2	-	-	250	-	-	0.5	1 × 1 × 500	1 × 1 × 250	125,250
10	FC_3	-	-	50	-	-	0.5	1 × 1 × 250	1 × 1 × 50	12,550
11	Output	-	-	5	Softmax	-	-	1 × 1 × 50	1 × 1 × 5	255

### Parameters

Hyperparameters are essential when developing a Convolutional Neural Network (CNN) since they determine the model’s effectiveness alongside efficacy. The Initial Learning Rate has been configured to 1 × 10^− 4^, indicating that rapidly the CNN Model adjusts to a given challenge throughout the training. The reduced learning rate may result in increased steady convergence, yet it takes longer epochs to achieve the best results. The Max Epochs option has been configured to 10, indicating that the CNN will be trained across the whole dataset at least ten times throughout training. The Shuffle parameter has been configured for “every-epoch,” guaranteeing that the input dataset will be chosen randomly before the start of every single epoch, helping to prevent the CNN model from learning any sequence-dependent patterns. The Validation Frequency has been configured to 30, which means that the model’s efficiency on a validation set is evaluated every 30 iterations, enabling overfitting to be continuously monitored throughout the train. The Mini-Batch Size has been configured to 64, indicating the amount of input data that will have to be routed across the CNN model when the CNN weights are modified. This size strikes a compromise between rapidity and precision in gradient assessment. Lastly, the optimizer employed is Stochastic Gradient Descent with Momentum (SGDM), which expedites convergence and smooths out oscillations by considering both the gradient and momentum of the preceding stages. Such hyperparameter settings collaborate to optimize the CNN model.

To guarantee an equitable and repeatable comparability among every approach, we employed a standardized training process and established a predetermined computational resource. Models were trained using identical data preparation and augmentation strategies, optimization parameters, and assessment protocols. Table 2 comprehensively enumerates every setting and configuration, including data splits, image size and color management, normalization, augmentation policy, optimizer and hyperparameters, learning rate configuration, batch size, epochs, regularization, initialization, class imbalance management, random seeds, and model selection criteria. Any modifications made from this configuration are fully documented in the ablation research.

**Table 2:** Comprehensive hyperparameter settings and training parameters used in all the models.

Setting	Parameters
Mini-batch size	64
Momentum	0.9
L2Regularization	1e-4
MaxEpochs	30
Shuffle	Every-epoch
GradientThresholdMethod	l2norm
Optimizer	SGDM
InitialLearnRate	1 × 10⁻⁴
Augmentations	Flip (H/V), rotation ± 10°, translation ± 5%, brightness/contrast ± 10%

### Transfer learning

Transfer learning effectively uses pre-trained deep learning models by reusing models trained on many images. This method is usually used when images are scarce, as the advantages of this method are that it saves time in training and that developing an algorithm from scratch requires many images for training and testing^26^. Therefore, scientists resort to this method, focusing only on the layers in which the change is made.

#### ResNet

**Fig. 4:**
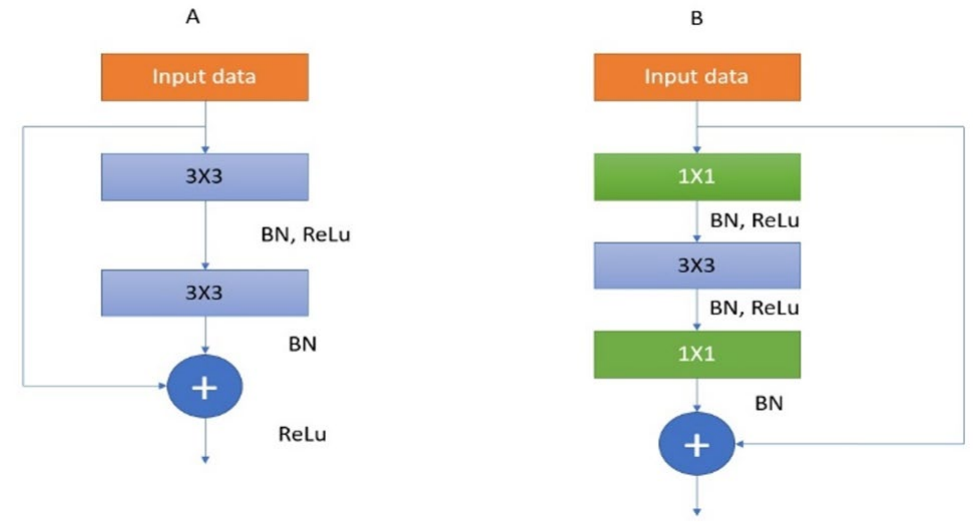
Architecture using a bottleneck residual module in ResNet18 is described in further detail in the reference^28^.

The Deep Residual Network (ResNet) is a widely recognized approach in Deep Learning, introduced by He et al.^27^. At the forefront of the CNN designs, this particular design stands out because it can validate network depth’s positive impact on visual representations, including various sorts of 152 layers. However, the model encounters 2 fundamental challenges when it is implemented for training to increase its depth. Each efficiency decreases, and vanished gradients are observed. To address such issues, ResNet incorporates a skip connection to mitigate the loss of information as the network becomes deeper. The primary concept underlying the construction of ResNet is the residual module, as depicted in Fig. 4. The left half of Fig. 4a displays two convolutional layers. 3 × 3 kernels were employed, and the spatial dimensions were maintained in addition. The right side uses a technique employed in the ResNet18 model to bypass connections by combining the input and output. In Fig. 4b, the input data is passed through two convolutional networks using kernel sizes of 1*1 and 3*3, also called bottleneck residuals. Various residual modules were employed. ResNet18 designs, the appropriate approach is to employ a skipping connection, which links the module’s input to an additional procedure to the outcome on the left path. The deep residual network is constructed by iteratively combining several residual modules and utilizing numerous traditional convolution and pooling layers. For our research, we utilized ResNet18.

#### GoogLeNet

**Fig. 5:**
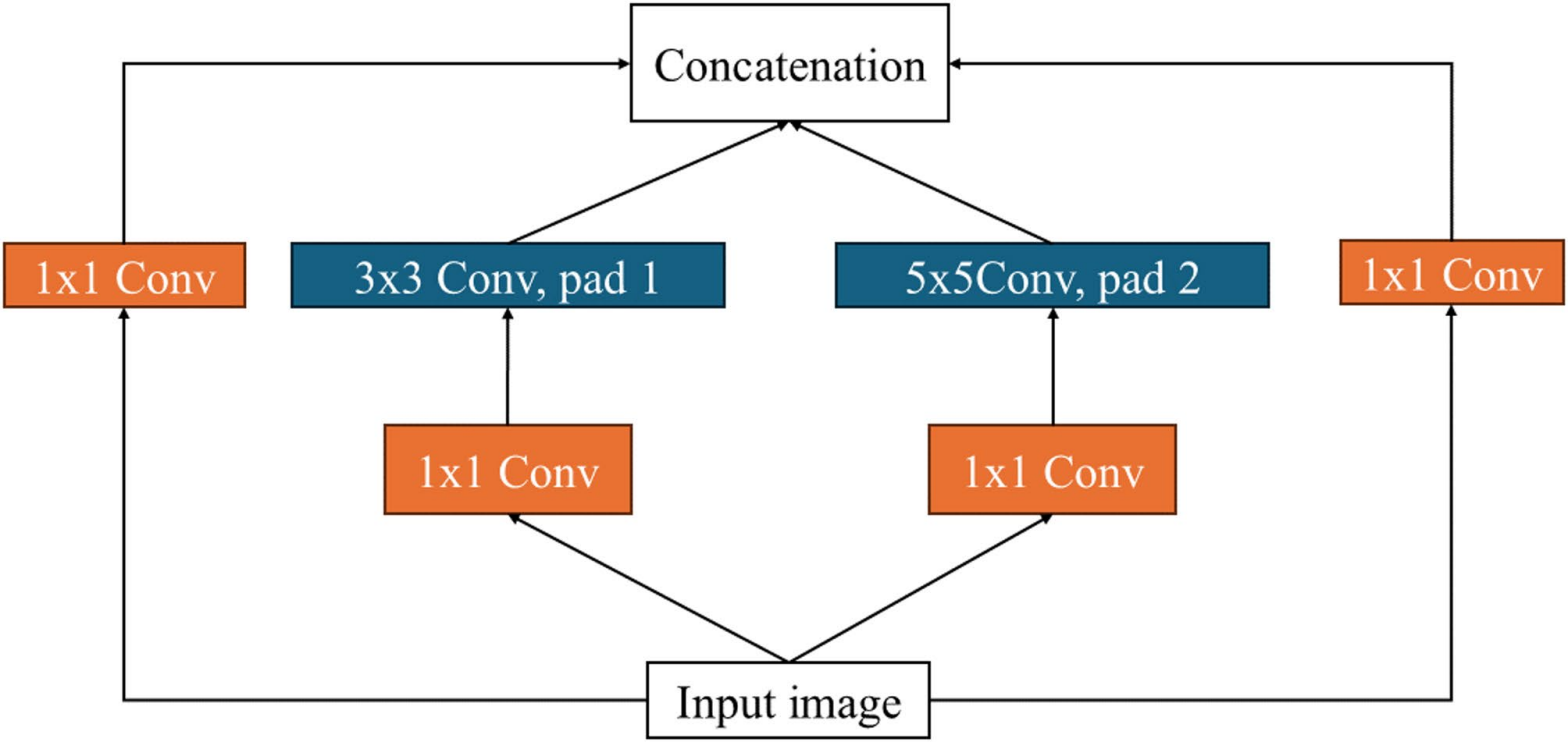
The GoogLeNet architecture^30^.

GoogLeNet’s architecture, which includes 22 layers, is responsible for its outstanding efficiency in picture recognition^29^. The infrastructure has 27 pooling layers, with 9 inception blocks arranged sequentially. Every inception block consists of four concurrent routes, and its conclusion is linked to the global average pooling layer. Figure 5 clarifies GoogLeNet’s structure.

#### MobileNet

MobileNetV2 is an enhanced version based on the existing MobileNetV1 model. It is the cutting-edge technology in Deep learning Convolutional Neural Networks (CNN). It is highly efficient for extracting features (employing DeepLabv3, which provides precision parameters for analyzing feature maps). The essential component utilized in this architecture consists of (1) straight bottlenecks located across the stages, plus (2) quick links present across the bottlenecks. The model’s structure is well-suited to accomplish the diverse results using various input images. MobileNetV2 model structures have 53 tiers and 3.5 million variables, using a starting imagine dimensions of 224 × 224 (width multiplier)^31^.

#### ShuffleNet

ShuffleNet is a highly effective convolutional neural network structure specifically developed to offer a favorable trade-off across quickness and precision. It is highly suitable for mobile and integrated platform applications. Through group convolutions and channel shuffling, ShuffleNet aims to minimize computing expenses whilst sacrificing effectiveness. Group convolutions enable the neural network to handle several pathways independently, minimizing the needed computations. The channel shuffling effectively facilitates data interchange among several groups, preserving the network’s ability to convey data. The amalgamation of these factors enables ShuffleNet to achieve superior precision while using fewer variables and computations than conventional neural networks. As a result, it is often used in contemporaneous fashion workloads with limited computing capabilities.

### Evaluation metrics

In this research, several metrics were used to verify the performance of the developed model based on the results obtained from the model (confusion matrix)^32^. After training and testing the model 30 times, the test results were used to evaluate the model in terms of accuracy, sensitivity, and specificity. Accuracy is calculated as the number of accurate predictions in the entire data set, as shown in Eq. 1. Likewise, sensitivity is calculated based on the positive results obtained from the data set and shown in Eq. 2. As for confidence, it is represented by the correct number obtained with positive predictions and is calculated through Eq. 3. Finally, the F1-Score is calculated by combining accuracy and recall, as using this criterion provides an ideal impression of the model and the results obtained. Similarly, Eq. 4 depicts the precision (Positive Predictive Value (PPV)), representing the ratio of true positive predictions to total positive predictions. The negative predictive value (NPV) is provided in Eq. 5. The Harmonic mean, often referred to by the F1-score, is calculated using precision and sensitivity, as given in Eq. 6. Lastly, the correction coefficient is calculated using the confusion matrix values and Matthew’s correlation coefficient range (MCC) from Eq. 7.

1 $$\:Accuracy\:=\frac{TP+TN}{TP+FP+TN+FN}\:$$

2 $$\:Recall=\mathrm{S}\mathrm{e}\mathrm{n}\mathrm{s}\mathrm{i}\mathrm{t}\mathrm{i}\mathrm{v}\mathrm{i}\mathrm{t}\mathrm{y}=\frac{TP}{TP+FN}$$

3 $$\:Precision=Confidence=\frac{TP}{TP+Fp}$$

4 $$\:F1-score=\frac{2*TP}{2*TP+FP+FN}$$

5 $$\:\mathrm{n}\mathrm{e}\mathrm{g}\mathrm{a}\mathrm{t}\mathrm{i}\mathrm{v}\mathrm{e}\:\mathrm{p}\mathrm{r}\mathrm{e}\mathrm{d}\mathrm{i}\mathrm{c}\mathrm{t}\mathrm{i}\mathrm{v}\mathrm{e}\:\mathrm{v}\mathrm{a}\mathrm{l}\mathrm{u}\mathrm{e}\:\left(NPV\right)=\frac{TN}{TN+FN}$$

6 $$\:F1-score=\frac{2*TP}{2*TP+FP+FN}$$

7 $$\:MCC=\frac{\left(TP*TN\right)-\left(FP*FN\right)}{\sqrt{(TP+FP)(TP+FN)(TN+FP)(TN+FN)}}$$

##  Results and discussion

This section will explain the results obtained by developing and training an algorithm to classify bacteria based on images obtained using a confocal microscope. There is a distinct separation between E. coli and S. aureus, as indicated by the fact that the per-run 2 × 2 confusion matrix, which consists of 30 repetitions with a 5-fold every one, is extremely diagonal. It is considerably more frequently that S. aureus is incorrectly identified as E. coli compared to the other way around, which reflects a significantly greater recall for E. coli and a little lower detection for S. aureus. Non-diagonal numbers are continuously small and possibly asymmetrical. We place an emphasis on standardized levels in the aggregated matrix since class totals remain consistent from run to run, given that mild E. coli constitutes the majority. The matrix values, when taken as a whole, demonstrate that the majority of errors cluster across a single off-diagonal, and there is no indication of mode collapse or fold-specific instabilities. This provides validation for the reliability of the structure, alongside the tight variation that was observed in the primary findings.

The results are attached in the appendix files for all the results obtained. As for the results attached in Table 3, the best result was for R12, as it was the best result obtained, in addition to the fewest misclassifications = 227 (98 + 129). As for R16, it achieved the lowest results, with an error rate of 374 misclassifications. Moreover, this pattern is supported by the aggregated versions of the 5-fold confusion matrices for each of the 4 pre-trained models: When it comes to non-diagonal numbers, ResNet18 has the lowest number (198 E. coli and S. aureus and 108 S. aureus and E. coli; 306 overall; 95.67% correctness). ShuffleNet comes in second with 241,89; 330 overall; 95.33%, after MobileNetV2 (319,84; 403 overall; 94.30%), and GoogLeNet has the lowest performance (347,178; 525 overall; 92.57%).

**Table 3:** Confusion matrix results for algorithms used in classifying bacteria.

Feature name	Classes name		5-K	
			Predicted class	
GoogLeNet	Actual Class	E.coli	3664	347
		S.aureus	178	2877
Mobilenetv2	Actual Class	E.coli	3758	319
		S.aureus	84	2905
ResNet18	Actual Class	E.coli	3734	198
		S.aureus	108	3026
ShuffleNet	Actual Class	E.coli	3753	241
		S.aureus	89	2983
**CM-Net**	**Actual class**	**E.coli**	**3713**	**98**
		**S.aureus**	**129**	**3126**

The convolutional neural network was the backbone of the proposed model for this method. l, the developed model was trained and tested extensively more than 30 times (using 5 K cross-validation each time it was trained and tested with a ratio of 80 − 20) to ensure the robustness and reliability of the model. This leads to a comprehensive examination of the model, greater assurance of accurate results, and high efficiency. In terms of accuracy, the results show that the proposed model achieved results higher than 94.78% in its lowest case, which was in Round No. R24. The highest results were in Round 12, which was 96.79%. The results obtained ranged from 94% to 96%, indicating that the model achieved slightly different results and was not biased towards one of the classes. The average results were calculated for all criteria used and shown in Table 4 in bold. Based on the results, the proposed method demonstrates the possibility of using this model to classify bacteria.

**Table 4:** Delineates the findings from the training and testing, offering a thorough summary of the outcomes within different types of measurements (in percentage).

Rs	Acc	Sen	Spe	Pre	NPV	F1-score	MCC	Time
R1	96.59	96.88	96.25	96.85	96.28	96.86	93.13	7.078
R2	96.39	96.43	96.34	96.91	95.78	96.67	92.73	9.5467
R3	96.32	95.94	96.77	97.26	95.24	96.59	92.60	9.4265
R4	94.91	95.29	94.45	95.34	94.39	95.31	89.73	8.8375
R5	95.97	95.13	96.96	97.39	94.36	96.25	91.92	7.9525
R6	96.24	96.77	95.60	96.32	96.13	96.55	92.41	9.8489
R7	96.73	96.88	96.56	97.10	96.29	96.99	93.41	8.1009
R8	95.80	94.85	96.93	97.36	94.04	96.08	91.59	10.2930
R9	96.32	95.81	96.93	97.38	95.10	96.59	92.61	8.8431
R10	96.69	96.95	96.37	96.95	96.37	96.95	93.33	8.5645
R11	96.59	96.85	96.28	96.88	96.25	96.86	93.13	7.3217
R12	96.79	96.64	96.96	97.43	96.04	97.03	93.53	8.5539
R13	96.38	95.78	97.08	97.51	95.08	96.64	92.73	10.0540
R14	96.35	96.15	96.59	97.11	95.46	96.63	92.65	8.6314
R15	96.18	96.43	95.87	96.53	95.76	96.48	92.30	9.5277
R16	94.71	94.27	95.22	95.92	93.31	95.09	89.37	8.4402
R17	96.05	95.94	96.18	96.77	95.21	96.35	92.05	8.0613
R18	96.60	96.67	96.53	97.07	96.05	96.87	93.16	8.6468
R19	96.12	96.33	95.87	96.53	95.64	96.43	92.19	9.9228
R20	95.92	95.52	96.40	96.94	94.76	96.22	91.81	10.0168
R21	94.99	94.82	95.19	95.92	93.91	95.37	89.92	7.5133
R22	96.43	95.86	97.12	97.54	95.17	96.69	92.84	9.4124
R23	96.28	96.41	96.12	96.74	95.74	96.57	92.50	10.5476
R24	94.78	95.11	94.39	95.28	94.18	95.19	89.48	8.2316
R25	96.21	95.89	96.59	97.10	95.17	96.49	92.37	9.9285
R26	95.77	95.63	95.94	96.56	94.85	96.09	91.48	9.4124
R27	96.42	96.98	95.75	96.45	96.38	96.72	92.78	7.4475
R28	96.43	96.59	96.25	96.84	95.95	96.72	92.81	9.0993
R29	95.16	94.74	95.66	96.30	93.85	95.51	90.27	11.0555
R30	96.38	96.02	96.81	97.28	95.33	96.65	92.72	8.9215
Average	96.08	95.98	96.19	96.78	95.26	96.38	92.11	8.97459

Several popular deep learning models were used for transfer, which are widely used in image data classification. Specifically, there are four different models: GoogLeNet, MobileNetV2, ResNet18, and ShuffleNet. We selected these models because of their high image recognition capabilities and the diversity of each one’s architecture. The results obtained were compared with the developed model for bacterial classification. Remarkably, the developed model achieved higher results than the four models in all metrics. This indicates that the developed model can highly classify microorganisms accurately. As detailed in Table 5, the overall results show the results obtained. In comparison to ShuffleNet (2.3 M), ResNet-18 (3.5 M), GoogLeNet (6.8 M), and MobileNetV2 (11.7 M), CM-Net is the algorithm with the fewest parameters, with 1.68 M. It is also the architecture with the least amount of space. Besides its good accuracy, the design demonstrates the proposed model’s ability to reduce memory usage and computational costs. This balance allows the model to be used for operations even on resource-constrained platforms.

**Table 5:** Comparison of CM-Net with commonly employed models that have undergone training (in percentage).

Rs	Acc	Sen	Spe	Pre	NPV	F1-Score	MCC	Time	Parameters (M)
GoogLeNet	92.57	95.37	89.24	91.35	94.17	93.31	85.06	46.1681	6.8
Mobilenetv2	94.30	97.81	90.11	92.18	97.19	94.91	88.64	19.9435	11.7
ResNet18	95.67	97.19	93.86	94.96	96.55	96.06	91.28	25.9159	3.5
ShuffleNet	95.33	97.68	92.52	93.97	97.10	95.79	90.64	105.803	2.3
**CM-Net**	**96.08**	**95.98**	**96.19**	**96.78**	**95.26**	**96.38**	**92.11**	**8.97459**	**1.68**

We performed a selective ablation, maintaining all other variables constant (group-wise splits, preprocessing/normalization, augmentation policy, optimizer, learning rate schedule, batch size, and total update budget). Regarding every variety, we macro-averaged class-specific metrics (E. coli, S. aureus) to present a class-balanced perspective. The findings indicate that a moderate structural capability close to (AddConvolutional6) produces the greatest comparable gains among measures (about + 3.0% on average, including a significant enhancement in MCC), succeeded by a slight width augmentation (WideX2, approximately + 2.8%). Substituting the clipped activation with a conventional ReLU yields a modest yet consistent advantage (about + 0.7% points). Conversely, eliminating Batch Normalization as well as adopting the alternative optimizer method (SGDA) both diminish efficiency (by approximately − 2.8% and − 2.3% on average), highlighting the stabilization effect of BN and the significance of the selected optimization configuration. Collectively, our findings identify the factors that directly influence generalization according to a carefully defined training budget and augmentation strategy, demonstrating applicability among several classes instead of preferring a singular classification. All results obtained by systematic ablations are included in the Supplementary (ablation results table S1 and S2), which details all the results obtained.

When examining the raw effectiveness, CM-Net exhibits a greater amount of the characteristics of a steady biological reflex—precise, consistent, and balanced—than a typical Deep learning model. The accuracy is approximately 96%, with minimal fluctuation across iterations, while sensitivity (the capacity to accurately identify the positive class) and specificity (the capacity to precisely exclude the negative class) are closely aligned without negatively affecting each other; the other competing designs fail to attain that symmetry. Other models, such as MobileNetV2 and ShuffleNet, have shown significantly decreased essential effectiveness and reduced reliability. This implies that their efficacy depends on specific data sampling, which limits their use as a reliable underlying decision boundary. Even when the median correctness of a traditional algorithm, like ResNet18, approaches that of CM-Net, it’s doing so with a wider confidence interval and lower dependability in downstream, applicable measures like the Matthews correlation coefficient (MCC): CM-Net achieves an average classification accuracy in the lower 90s, compared to ultralight pre-trained model around the 80s, indicating that CM-Net not only correctly identifies numerous samples but also ensures balanced concordance among predicted and actual labels across both classes. All the results of the model are illustrated in Table 6.

**Table 6:** The overall evaluation of CM-Net performance with respect to deep convolutional core networks (GoogLeNet, ResNet-18, MobileNetV2, and ShuffleNet) shows consistent gains in diagnostic accuracy, statistical reliability, and model robustness.

Metric	CM-Net	GoogLeNet	ResNet18	MobileNetV2	ShuffleNet
Accuracy	96.04 ± 0.67 [95.21–96.87]	92.77 ± 2.25 [90.41–95.13]	95.03 ± 2.18 [92.32–97.74]	89.97 ± 7.84 [80.23–99.71]	89.74 ± 1.09 [88.38–91.09]
Sensitivity	95.93 ± 0.74 [95.01–96.86]	93.73 ± 2.45 [91.17–96.30]	94.20 ± 2.28 [91.9–97.5]	89.50 ± 8.15 [79.37–99.62]	89.63 ± 1.10 [88.27–90.99]
Specificity	96.15 ± 1.00 [94.92–97.39]	92.04 ± 2.64 [89.28–94.79]	94.80 ± 2.28 [91.9–97.6]	89.50 ± 8.15 [79.37–99.62]	89.63 ± 1.10 [88.27–90.99]
Precision	96.75 ± 0.82 [95.73–97.77]	92.90 ± 1.92 [90.83–94.97]	95.20 ± 2.09 [92.61–97.08]	90.69 ± 7.63 [81.22–100.16]	89.70 ± 1.08 [88.35–91.04]
NPV	95.21 ± 0.85 [94.16–96.26]	93.70 ± 2.22 [91.35–96.06]	95.00 ± 2.90 [92.10–97.80]	90.69 ± 7.63 [81.22–100.16]	89.70 ± 1.08 [88.35–91.04]
F1-score	96.34 ± 0.61 [95.57–97.10]	93.22 ± 2.22 [90.87–95.58]	94.97 ± 2.22 [92.21–97.72]	89.73 ± 8.04 [79.74–99.72]	89.65 ± 1.10 [88.29–91.01]
MCC	92.02 ± 1.35 [90.34–93.70]	85.52 ± 4.58 [80.47–90.56]	90.02 ± 4.37 [84.60–95.45]	80.17 ± 15.73 [60.64–99.70]	79.32 ± 2.17 [76.62–82.02]

According to an analytical point of view, comparison differences offer a robust assessment of coherence and reliability of statistical data among structures. CM-Net exhibits significant percentage-point improvements over GoogLeNet in essential diagnostic measurements, including accuracy, sensitivity, precision, and F1-Score, bolstered by substantial associated impacts. This sequence of events demonstrates that CM-Net’s superiority is not due to random variation, but rather to consistent, paired performance. The disparity increases significantly when juxtaposed with lightweight architectures like ShuffleNet and MobileNetV2, as CM-Net outperforms the others by roughly ( 6–7) % in accuracy, sensitivity, F1-Score, and particularly in MCC. Remarkably substantial effects are linked to these variations, indicating that training structure has a big benefit rather than an incremental number of layers. The importance of these differences remains (q-values = 0), highlighting the durability of CM-Net’s efficiency. The comparison between CM-Net and ResNet18 is minimal, and generally about 1% with reduced effect sizes, suggesting that ResNet18 nears CM-Net’s performance threshold without matching its efficacy. The outcomes demonstrate a separate order of ability: CM-Net establishes the superiority, with ResNet18 as the closest competitor, GoogLeNet trailing at a discernible distance, while smaller alternatives significantly underperform. All the model’s differences are illustrated in Table 7.

**Table 7:** Comparison differences to CM-Net regarding accuracy, sensitivity, specificity, precision, NPV, F1-Score, and MCC, presented as Δ along bootstrap 95% CIs, false discovery rate-adjusted relevance (q), and paired effect sizes (Cohen’s d_z) with uniform data divisions.”

Metric	GoogLeNet (Δ; q; d_z)	ResNet18 (Δ; q; d_z)	MobileNetV2 (Δ; q; d_z)	ShuffleNet (Δ; q; d_z)
Accuracy	+ 3.27 [+ 1.61–+5.58]; q = 0.081; d_z = 1.20	+ 1.01 [-0.45–+2.59]; q = 0.426; d_z = 0.515	+ 6.06 [+ 0.10–+12.77]; q = 0.213; d_z = 0.738	+ 6.30 [+ 5.80–+6.97]; q = 0.000; d_z = 8.501
Sensitivity	+ 2.20 [+ 0.92–+4.68]; q = 0.081; d_z = 1.14	+ 1.11 [-0.74–+2.97]; q = 0.426; d_z = 0.457	+ 6.44 [+ 0.34–+13.40]; q = 0.213; d_z = 0.760	+ 6.31 [+ 5.60–+7.02]; q = 0.000; d_z = 6.926
Specificity	+ 4.11 [+ 2.33–+6.52]; q = 0.081; d_z = 1.28	+ 1.33 [+ 0.14–+2.80]; q = 0.426; d_z = 0.801	+ 6.66 [+ 0.13–+13.37]; q = 0.213; d_z = 0.772	+ 6.53 [+ 5.80–+7.41]; q = 0.000; d_z = 6.167
Precision	+ 3.85 [+ 1.92–+6.32]; q = 0.081; d_z = 1.32	+ 1.55 [+ 0.41–+2.88]; q = 0.426; d_z = 0.994	+ 6.06 [+ 0.50–+12.67]; q = 0.213; d_z = 0.763	+ 7.05 [+ 6.40–+7.75]; q = 0.000; d_z = 8.085
NPV	+ 1.51 [+ 0.18–+3.44]; q = 0.109; d_z = 0.91	+ 0.01 [-1.78–+1.70]; q = 0.995; d_z = 0.003	+ 4.52 [-0.73–+11.50]; q = 0.280; d_z = 0.558	+ 5.51 [+ 4.86–+6.10]; q = 0.000; d_z = 6.787
F1-score	+ 3.12 [+ 1.58–+5.48]; q = 0.081; d_z = 1.27	+ 1.37 [-0.14–+2.99]; q = 0.426; d_z = 0.680	+ 6.61 [+ 0.48–+13.42]; q = 0.213; d_z = 0.789	+ 6.69 [+ 6.16–+7.37]; q = 0.000; d_z = 8.646
MCC	+ 6.50 [+ 3.14–+11.27]; q = 0.081; d_z = 1.18	+ 2.00 [-0.90–+5.15]; q = 0.426; d_z = 0.514	+ 11.86 [+ 0.12–+25.49]; q = 0.213; d_z = 0.720	+ 12.70 [+ 11.72–+14.05]; q = 0.000; d_z = 8.534

Validity analysis confirms that we used CM-Net as the benchmark. While comparing CM-Net to ShuffleNet, the p-values for nearly all fundamental metrics—accuracy, sensitivity, specificity, precision, F1-Score, and MCC—are within limits typically regarded to be highly important, suggesting that the demonstrated higher accuracy is unlikely attributable to random variation. In comparison to GoogLeNet, the paired t-tests generally hover near traditional decision thresholds: they are sufficiently low to indicate that CM-Net is consistently superior. In comparison to ResNet18, the p-values increase, indicating an empirical interaction between the two models: CM-Net generally prevails; however, the disparity is nuanced instead of striking. All the model’s paired t-tests are illustrated in Table 8.

**Table 8:** Comparative analysis for CM-Net within standardized assessment: Two-sided paired t-test p-values describing for each metric disparities relative to 4 primary pretrained models throughout repeated, split-consistent executions.

Metric	GoogLeNet (t *p*)	ResNet18 (t *p*)	MobileNetV2 (t *p*)	ShuffleNet (t *p*)
Accuracy	0.056	0.313	0.174	0.002
Sensitivity	0.061	0.365	0.164	0.009
Specificity	0.048	0.148	0.159	0.001
Precision	0.042	0.090	0.163	0.001
NPV	0.110	0.995	0.280	0.011
F1-score	0.045	0.203	0.153	0.003
MCC	0.058	0.314	0.183	0.002

**Fig. 6:**
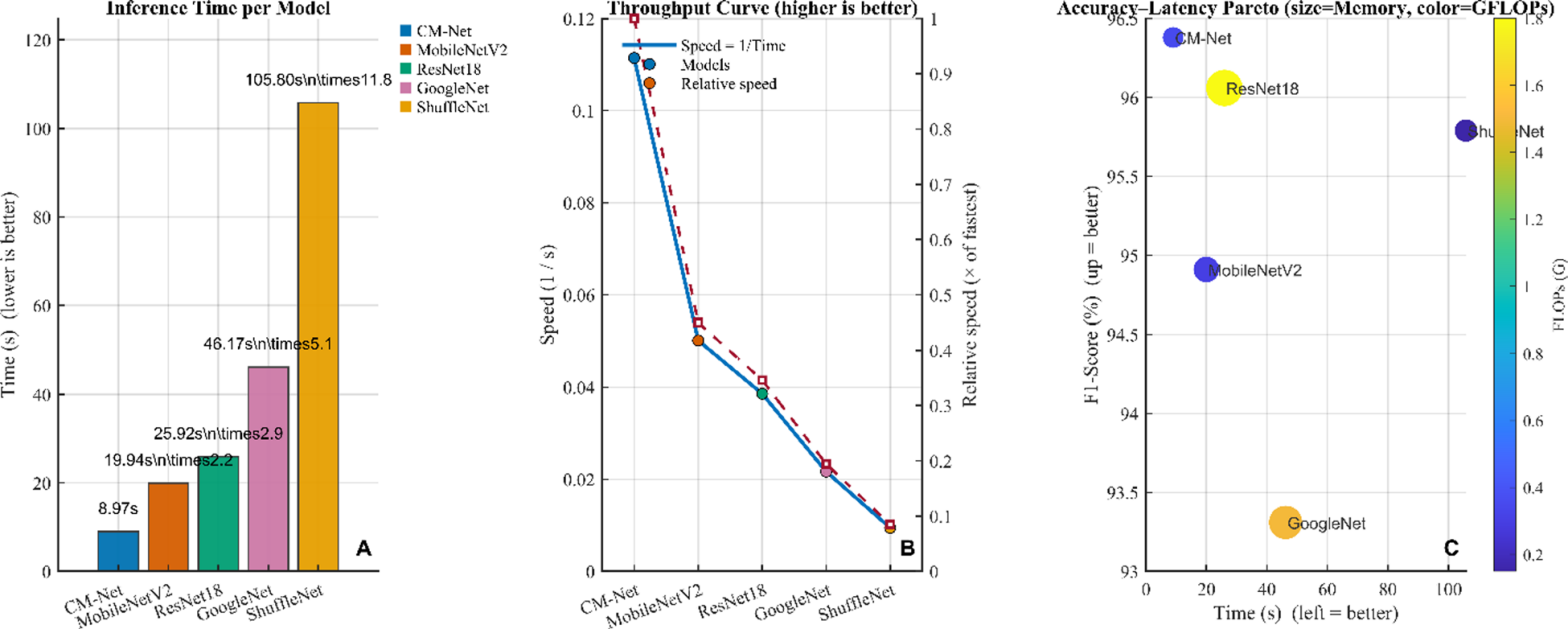
Comparative analysis of accuracy and effectiveness among algorithms. (**A**) Inference runtime for each algorithm (smaller values are preferable); bars indicate absolute delay along with the decrease in ratio compared to the quickest model, CM-Net (8.97 s). (**B**) Maximum throughput (1/latency; greater values are preferable) is depicted via a dashed curve representing efficiency standardized to CM-Net. Accuracy–latency Pareto: F1-score (%) compared to latency (s); bubble size represents memory (MB) and color indicates FLOPs (G). With uniform preparation, training resources needed, and hardware required, CM-Net occupies the Pareto frontier, integrating the quickest inference, the highest F1 score, and the minimal resource utilization.

A summary of the performance trade-offs for every structure is presented in Fig. 6. A CM-Net achieves the smallest latency (about 8.97 s), while MobileNetV2, ResNet-18, GoogLeNet, and ShuffleNet are around 2.2 times slower, 2.9 times slower, 5.1 times slower, and 11.8 times slower, correspondingly. As a result, B presents output as throughput (1/latency) and ensures the exact same ordering. Additionally, the speed relative curve has been standardized to CM-Net, which highlights similar differences among algorithms. C provides a contextualization regarding speed through demonstrating F1-score vs. latency while encoding memory and FLOPs. CM-Net represents the top-left quadrant alongside a smaller bubble and modest FLOPs, showing that it offers the highest accuracy–efficiency ratio. Based on our comparison, ResNet-18 is comparable to CM-Net in terms of F1; however, it has a latency that is almost three times greater and a bigger memory capacity. MobileNetV2 exhibits a moderate F1 score at a moderate latency, while GoogLeNet provides the smallest F1 score, and ShuffleNet is the slowest.

**Fig. 7:**
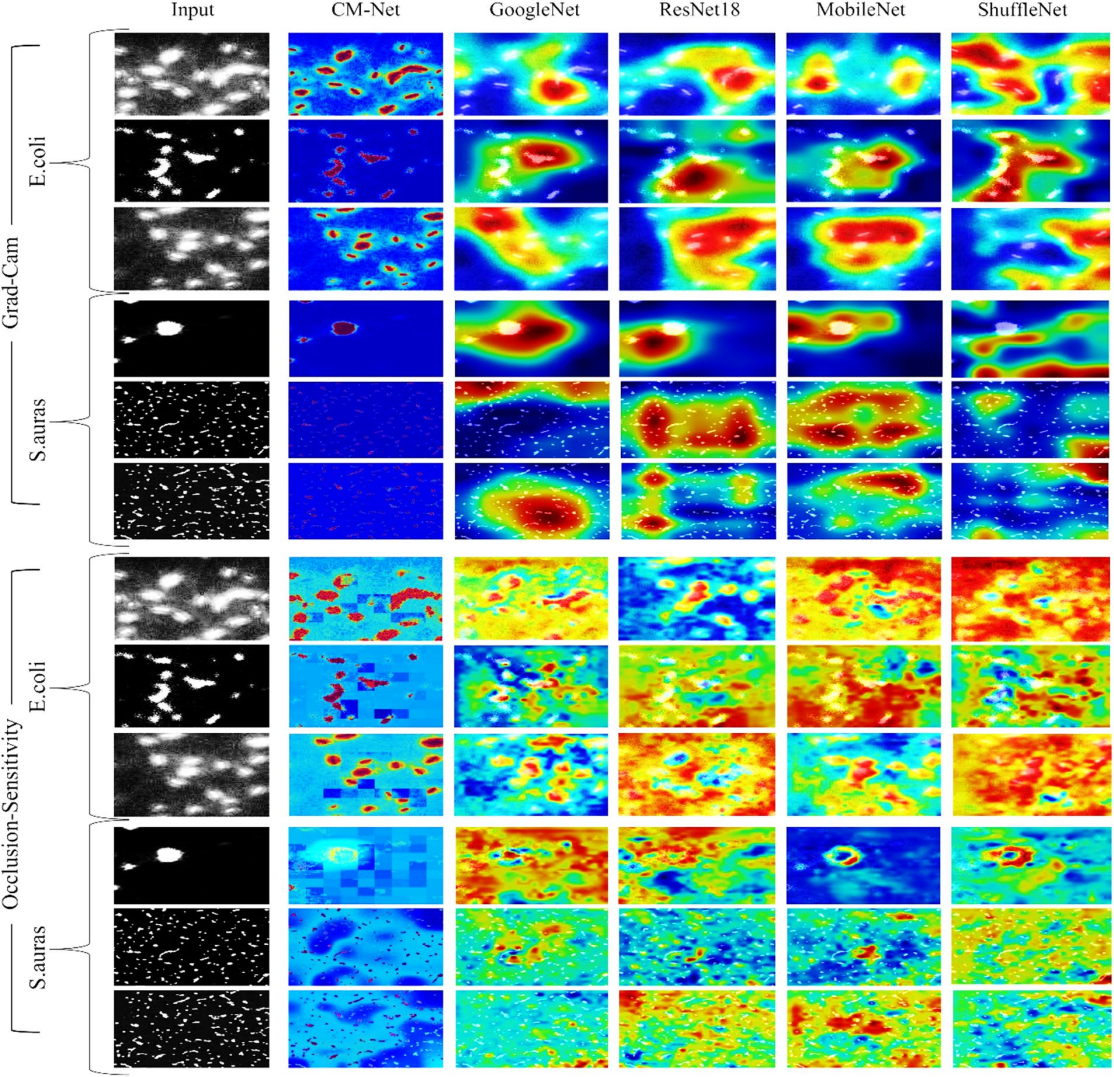
A demonstration of the CM-Net that effectively concentrated on the bacteria inside the images, as well as compared to the pre-trained model.

To perform the model intuitively, we used the Grand-CAM method, a visual tool that can detect the region the model has focused on and thus shows the places the model has strongly focused on. This method represents a visual method for the model and the ability to extract features from the image, affecting the classification results. CM-Net demonstrates substantial sensitivity towards bacterial bodies and their adjacent shadows in each E. coli and S. aureus; obscuring these patterns consistently diminishes trust, but masking surroundings or detritus has less impact. Conversely, the pre-trained model backbones frequently exhibit wider, dispersed sensitivity that merges with background speckle, indicating a partial dependence on accidental stimuli. The strong alignment among CM-Net’s occlusion maps and its Grad-CAM offers strong proof that the algorithm’s conclusions are based on biological shape instead of extraneous context, signifying reliable, biologically relevant interpretation. Figure 7 shows that the model focused well on the bacteria in the images.

To assess the interpretability of our designed CM-Net, we conducted a thorough analysis of Grad-CAM and Occlusion Sensitivity activation patterns against the actual bacterium areas by calculating Intersection over Union (IoU) and Dice coefficients. These measurements assess the positional correspondence between the model’s focus and important biological visual locations, therefore explaining the transparency and dependability of acquired interpretations. Table 9 illustrates that CM-Net attained the greatest average IoU (0.113) and Dice (0.309), significantly surpassing GoogLeNet (0.097, 0.161), MobileNet V2 (0.0915, 0.152), ResNet-18 (0.091, 0.142), and ShuffleNet (0.0595, 0.105). Such a benefit underscores CM-Net’s capacity to provide precise classification while preserving spatial coherence, as well as the biological significance and the relationship between gradient- and occlusion-based explainability approaches. Such uniformity indicates a profound understanding of bacterial morphology rather than mere texture identification, demonstrating that CM-Net prioritizes biologically significant information in confocal microscopy images.

**Table 9:** Quantitative evaluation of explainability effectiveness comparing CM-Net and pretrained models utilizing Grad-CAM and occlusion sensitivity measurements (IoU and dice coefficients) to examine the regional and biological applicability of models’ concentration regions in the bacterium identification of images.

Model	IoU_GC	IoU_OCC	Dice_GC	Dice_OCC	Avg_IoU	Avg_Dice
CM-Net	0.117	0.109	0.344	0.274	0.113	0.309
GoogLeNet	0.083	0.111	0.144	0.178	0.097	0.161
MobileNetV2	0.076	0.107	0.133	0.171	0.0915	0.152
ResNet18	0.079	0.103	0.137	0.147	0.091	0.142
ShuffleNet	0.075	0.044	0.133	0.077	0.0595	0.105

To ensure the calibration of the obtained results with numerous studies published in scientific journals, Table 10 shows that the proposed model achieves the highest scores, demonstrating superiority compared to previous results. This maintains transparency in reporting by providing detailed data. This ensures the reliability and integrity of this model, as well as its applicability in biological applications for highly accurate classification without affecting the results.

**Table 10:** Comprehensive evaluation of documented deep-learning techniques for biological microscopy: sample quality, experimental decisions, and statistical results (accuracy, precision, recall).

Ref.	Dataset	Samples	Method	Acc	Precision	Recall
^33^	Confocal microscopy	2000	ResNet50	81	81	75
^34^	Bacterial microscopy	1500	Multiple Deep learning	64	66	64
^35^	Confocal microscopy	3200	CNN	92		
^36^	microscopic images	1800	Deep learning	95	74	79
^37^	time-lapse microscopic images	1000	Deep neural network	80	NA	NA
^38^	Bacterial microscopy	2500	CNN	92	90	91
^39^	Spectroscopy	900	CNN	94	NA	NA
^40^	microscopic images	1200	Transfer Learning	92	NA	NA
^41^	Microscopic images	5000	YOLOv4	86	NA	NA
^42^	Self-prepared	800	LeNet	75	NA	NA
^43^	Pathogenic bacteria	3500	GoogLeNet	95	NA	NA
^44^	Pathogenic bacteria	2700	CNN	94	NA	NA
^45^	Self-prepared	6,000	CNN	95		
^46^	Bacterial microscopy	1,000	YOLO	67	73	74

The CNN structure, CM-Net, built during the current investigation is designed to overcome the constraints observed in pre-trained techniques like GoogLeNet, MobileNet, ResNet18, and ShuffleNet within identifying categories of microbiological datasets. While pre-trained algorithms may be successful in basic picture detection activities, their training on non-biological data sources such as ImageNet sometimes restricts their capacity to accurately identify specialized dataset forms, including microbiological microscope images. The fundamental constraint of transfer learning provided the rationale for developing a bespoke structure for this application. The innovative structure and characteristics of the construction of CM-Net involved utilizing five convolutional blocks, which comprised convolution layers, batch normalization, clipped reverse linear unit (ReLU) activation, and max-pooling layers. The unique aspect of this approach exists in the incorporation of clipped ReLU activation, which effectively avoids very high activation levels and addresses the issues of vanishing or exploding gradients. This facilitates the extraction of increasingly complex characteristics derived from microbiological pictures by deeper network layers. Once the depth of the neural structure expanded, the complexity of every convolutional block scaled up, enabling a progressive and efficient learning process. Such rigorous architecture enhanced the integrity plus effectiveness of the structure through the training process. The designs improved by including fully connected layers after the convolutional layers. Within these layers, dropout was implemented to mitigate overfitting. Dropout is a stochastic activation technique used after training to ensure that the algorithm applies consistently to new inputs. These comprehensively linked layers enabled the integration of obtained attributes and improved classification capability. Table 11 compares CM-Net with widely utilized models that have been trained, emphasizing the architectural choices made explicitly for microorganism identification of images.

**Table 11:** Compares CM-Net with several different types of pre-trained models in terms of architecture.

Feature	CM-Net	Pre-Trained
Purpose of design	Precisely developed for the classification of biological images,	Multipurpose Image Classification
Activation function	It is extremely important to *Clipped ReLU* to avoid vanishing or increasing gradients, which will enhance learning in layers of greater depth.	*ReLU* (standard),
Structure complexity	Architectural simplification: eliminates potentially costly elements	residual connections, depthwise separable convolutions, and inception modules.
Number of convolution blocks	5 convolutional blocks: Every block is meticulously designed for feature extraction in the microorganism dataset, utilizing typical convolution, batch normalization, clipped ReLU, and max-pooling layers.	GoogLeNet: 9 inceptions MobileNetV2: bottleneck ResNet18: residual ShuffleNet: 16 convolutions

**Fig. 8:**
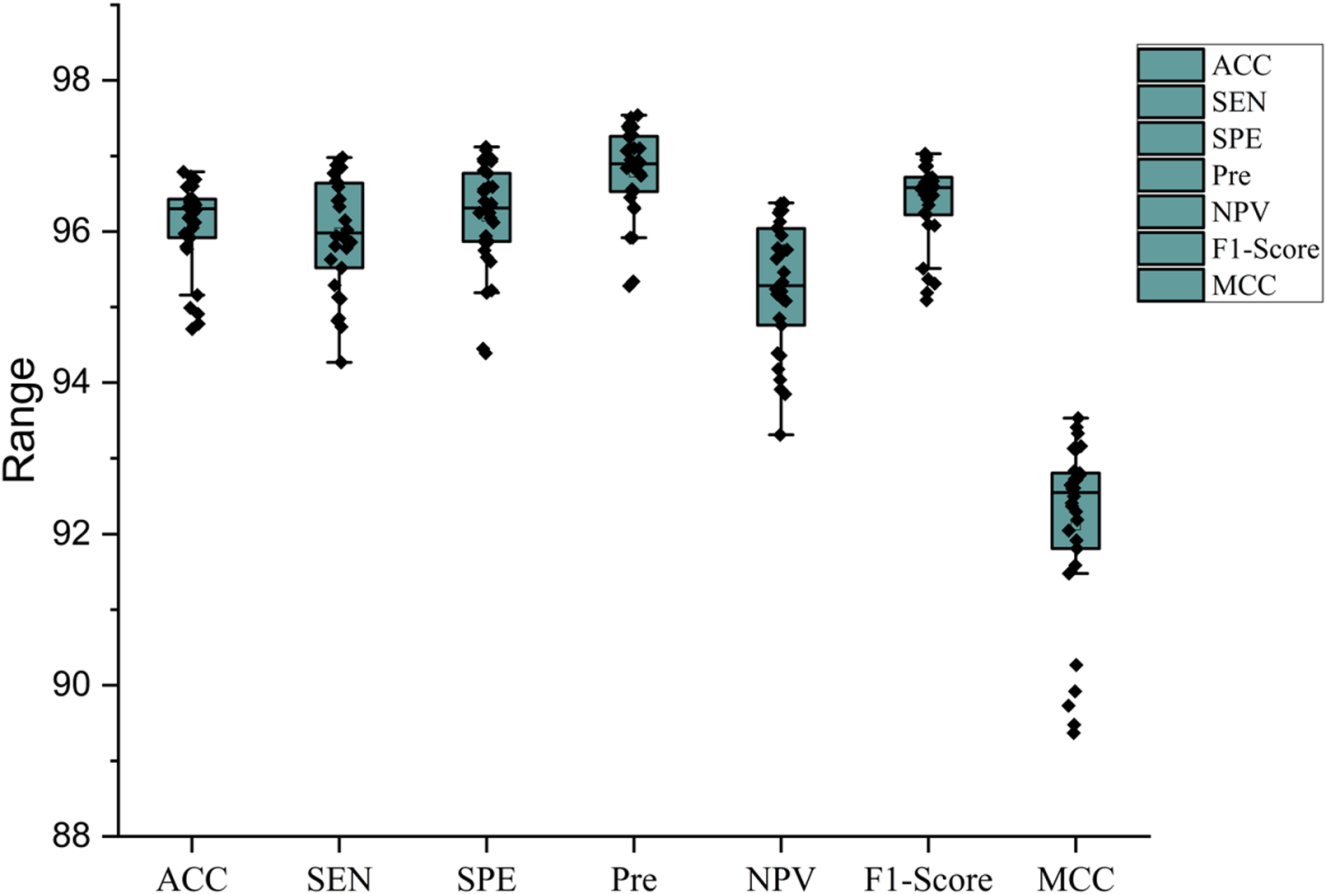
The boxplot of performance metrics for CM-Net through training and testing the model 30 rounds.

The findings achieved by the proposed convolutional neural network (CNN) design and training phase for bacterial classification demonstrate quite encouraging efficiency. The design was thoroughly evaluated after over 30 training rounds, including 5-fold cross-validation. By exposing the algorithm to rigorous evaluation on several data splits, the accuracy and dependability of the model are guaranteed, hence reducing the potential impact of unpredictability or overfitting on outcomes. The algorithm’s correctness consistently varied between 94.78% (in Round 24) and 96.79% (in Round 12) over all rounds, with an average accuracy of over 95%. Figure 8 illustrates the number of essential indicators of efficiency, including ACC, SEN, SPE, Pre, NPV, F1-score, and MCC across the evaluation of each round. The boxplot demonstrates the limited range of outcomes throughout several criteria, highlighting the consistency and reliability inherent in developing CM-Net models throughout several training rounds.

The restricted range of findings indicates that the predictive model is highly stable and consistently attains superior accuracy, regardless of the data partition or iteration. A slight variation of approximately 2% between the highest and lowest accuracy suggests that the method is unbiased toward each microbiological category. So, the consistent characteristics of outcomes illustrate the algorithm’s ability to be applied effectively over a wide range of bacterial classifications. The restricted range of success criteria minimizes the potential for bias in the categorization of various microbiological groups. Ensuring adequate classification is essential, particularly in microscopic applications. The extrapolation performances of the model have been confirmed using Grad-CAM visualizations, allowing for a clear understanding of the particular portions of the images that the CNN structure prioritizes throughout the procedure of classification. Such graphic illustrations indicate that CM-Net effectively concentrates on the microbiological areas, consequently confirming the model’s effectiveness in retrieving pertinent characteristics.

## Conclusion

In this study, a new deep-learning model (CM-Net) was developed to classify images of bacterium types obtained using a confocal microscope. Each image was divided into smaller sub-images with dimensions of 224 × 224 using an augmentation method, resulting in a total of 7,066 images across both classes. The model’s structure is meticulously designed with five convolutional blocks, each specifically trained to extract critical features from input images, significantly improving the final classification accuracy. CM-Net was trained and examined more than 30 times to obtain accurate, certain, and reliable results. The model was evaluated using seven metrics showing promising-outcomes: accuracy (96.08%), sensitivity (95.98%), specificity (96.19%), precision (96.78%), NVA (95.26%), F1-score (96.38%), and MCC (92.11%), demonstrating its robust performance across diverse criteria. Further, the developed model was compared with several counterpart available pre-trained models (GoogLeNet, MobileNetV2, ResNet18, and ShuffleNet, ), demonstrating superior outcomes in accuracy, reliability, and time efficiency. The superior performance of CM-Net in microbiological classification tasks can be attributed to its specifically developed, effective structure and customized feature extraction techniques specifically designed for bacterial data. In principle, CM-Net introduces a low-cost, precise, fast, and straightforward image processing technique utilizing CNNs. Adapting this CNN for such purposes can prove valuable in treatment strategies, educational applications, and microbiological research.

### Limitations and future work

Despite these remarkable findings, there are some limitations that should be recognized and considered in future studies. The most important limitations that were considered in this study were restricted to two types of bacteria (Escherichia coli and Staphylococcus aureus). Therefore, the study lacks generalizability across different microscope types, as well as different batches of bacteria or different bacterial strains, since the data were based on only two types of bacteria. Furthermore, due to the difficulty in obtaining external data using confocal microscopes, no external data demonstrating the model’s performance was used. Therefore, extensive validation encompassing diverse bacterial species, independent cohorts, and varied data (images) methodologies is crucial to confirm toughness and practical applicability.

For future work, we will drastically increase both the diversity and volume of data by using more classes and expanding the number of data points and categories (additional bacterial species, progressive viability/death states, and antibiotic exposure levels). This will involve:

- Increasing the number of classes used for input by differentiating bacterial viability states (dead/alive/damaged).
- Using the complete original images instead of using augmentation techniques (slicing images into segments).
- Collecting data from multiple data centers, which will facilitate generalization and application.

For the practical implementation, we will use a Graph Convolutional Neural Network (GCNN)) A method that relies on the number of nodes and the edges. We will use edge detection to identify bacteria in the image, then transform the identified bacteria into the desired shape. Next, we will evaluate the pixels within the selected shape using Otsu thresholding to identify the best pixels and discard unwanted ones, thus naturally preserving the geometry. Next, we will calculate an adjacency matrix to determine the relationship between each node and the number of edges that node needs to connect with. This information will then be fed into the GCNN model, which can help the model to classify and detect the bacteria.

## Data Availability

The datasets generated and/or analyzed during the current study are not publicly available but can be obtained from the author, Dr. Ahmed Al-Jumaili, upon reasonable request.
